# Application of blood biomarkers for the future anti‐Aβ antibody therapy in Japan

**DOI:** 10.1002/alz.084859

**Published:** 2025-01-09

**Authors:** Kensaku Kasuga, Tamao Tsukie, Kazuya Igarashi, Takanobu Ishiguro, Akinori Miyashita, Osamu Onodera, Takeshi Ikeuchi

**Affiliations:** ^1^ Department of Molecular Genetics, Brain Research Institute, Niigata University, Niigata, Niigata Japan; ^2^ Department of Molecular Genetics, Brain Research Institute, Niigata University, Niigata Japan; ^3^ Department of Neurology, Brain Research Institute, Niigata University, Niigata Japan; ^4^ Brain Research Institute, Niigata University, Niigata, Niigata Japan

## Abstract

**Background:**

With the approval of Lecanemab in Japan following the U.S., biomarker‐based diagnosis of AD will be required in daily practice. However, PET or CSF are limited by cost, low availability, and invasiveness. In addition, frequent MRI is required to monitor amyloid‐related imaging abnormalities (ARIA), an adverse event unique to anti‐Aβ antibodies. Blood biomarkers are expected to be inexpensive, less invasive, and acceptable for frequent testing in daily practice.

**Method:**

Of the cases analyzed for AD‐related CSF biomarkers for diagnostic purposes from Department of Neurology, Niigata University and related facilities, 217 plasma available cases were included. Of the 217 cases, 113 were clinically diagnosed as Alzheimer's clinical syndrome (ACS) and 104 as non‐ACS, including 6 cerebral amyloid angiopathy related inflammation (CAAri) cases. CSF Aβ42/40 ratio, phosphorylated tau (pTau181), neurofilament light chain (NfL), and plasma pTau181 and NfL were measured. According to the research framework, AD was determined when both CSF Aβ42/40 ratio (as A marker) and CSF pTau181 (as T marker) were positive. The ROC curve was used to verify the accuracy of plasma pTau181 in differentiating AD from non‐AD, and the accuracy of plasma NfL in differentiating AD from CAAri, which has been suggested to share a common pathology with ARIA.

**Result:**

Plasma pTau181 differentiated AD group (CSF A+T+) from non‐AD group with high accuracy (AUC = 0.867). Plasma pTau181 also differentiated A+ group from A‐ group with high accuracy (AUC = 0.913, sensitivity 89.7%, specificity 82.9%). When the low‐risk threshold was made more stringent (Sensitivity 90%, 95%, 100%), negative predict value increased (78.1%, 88.9%, 100%). When the high‐risk threshold was made more stringent (Specificity 90%, 95%, 100%), positive predictive value increased (78.1%, 88.9%, 100%). However, as a trade‐off, the number of the intermediate risk group increased (16%, 35%, 81%). Plasma NfL discriminated CAAri group from AD group (ACS and CSF A+T+) with high accuracy (AUC = 0.947).

**Conclusion:**

In Japanese clinical practice, plasma pTau181 is considered to be useful for detecting AD pathology, and is expected to be utilized for screening of anti‐Aβ antibody therapy. Plasma NfL may also be useful in screening for adverse events of anti‐Aβ antibodies.

